# Supplementary material for: Complex‐centric proteome profiling by SEC‐SWATH‐MS
Source: Mol Syst Biol. 2019 Jan 14;15(1):e8438. doi: 10.15252/msb.20188438 (PMC6346213; doi:10.15252/msb.20188438)
Supplement: Supplementary file 7 — Dataset EV6 [file MSB-15-e8438-s007.zip › feature_plots_bioplex/Q9BYD1.pdf]

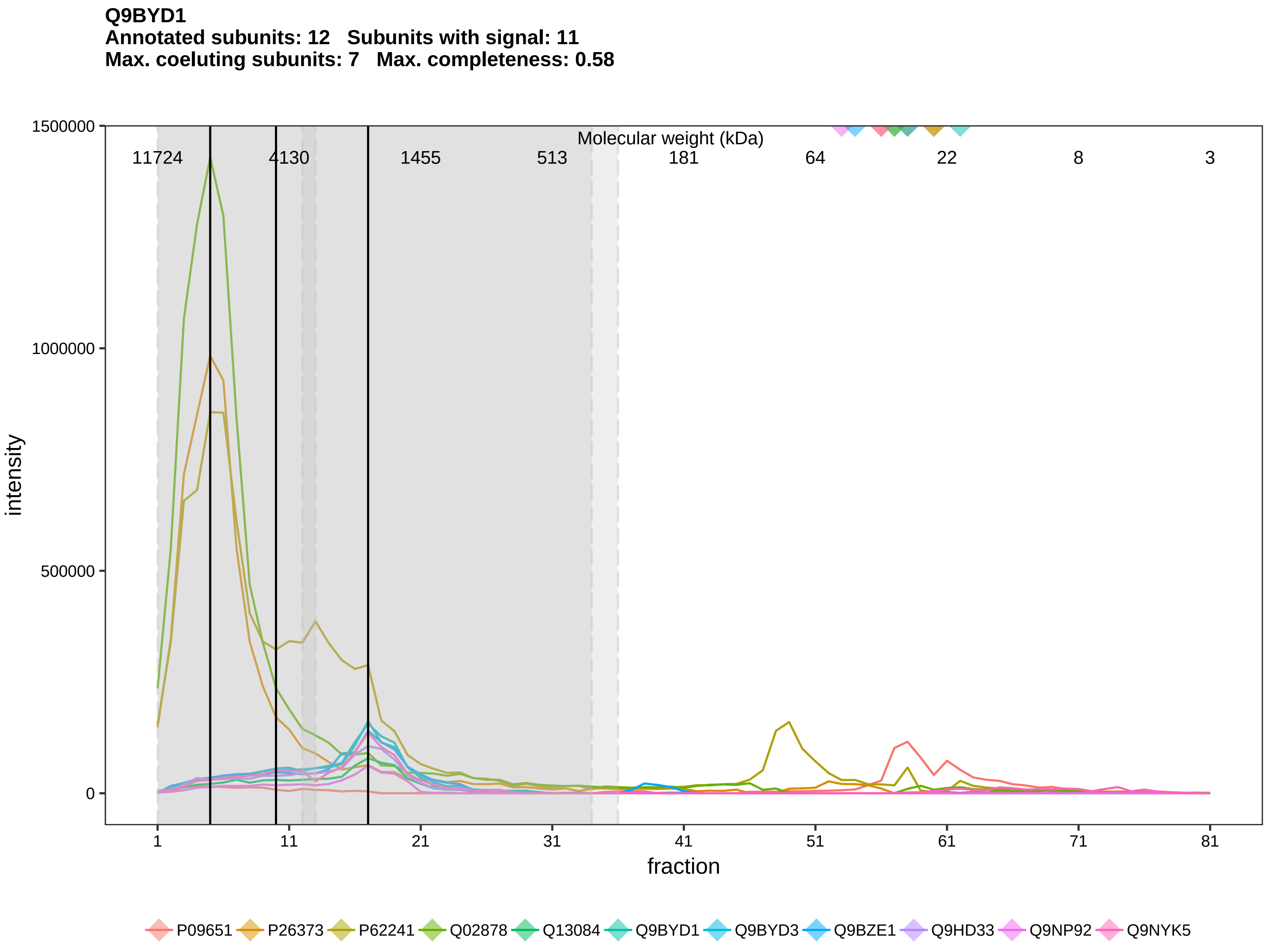

Q9BYD1
Annotated subunits: 12 Subunits with signal: 11
Max. coeluting subunits: 7 Max. completeness: 0.58
Molecular weight (kDa)
11724
4130
1455
513
181
64
22
8
3
intensity
1500000
1000000
500000
0
fraction
1
11
21
31
41
51
61
71
81
P09651
P26373
P62241
Q02878
Q13084
Q9BYD1
Q9BYD3
Q9BZE1
Q9HD33
Q9NP92
Q9NYK5
